# SOCIAL ISOLATION AND MENTAL HEALTH CHALLENGES AMONG HIGH-NEEDS VETERANS

**DOI:** 10.1093/geroni/igz038.1011

**Published:** 2019-11-08

**Authors:** Stuti Dang, Kiranmayee Muralidhar, Kaicheng Wang, Diana Ruiz, Carlos Gomez-Orozco, Willy Marcos Valencia

**Affiliations:** 1 Miami Veterans Affairs Healthcare System- GRECC, Miami, Florida, United States; 2 Department of Public Health Sciences, University of Miami Miller School of Medicine, Miami, Florida, United States; 3 Miami Veterans Affairs Healthcare System- Research Service, Miami, Florida, United States; 4 South Florida Veterans Affairs Foundation for Research and Education Miami, Miami, Florida, United States

## Abstract

Using predictive analytic modeling, the Veterans Affairs has identified vulnerable Veterans, labeled as High Need High Risk (HNHR), as those who need greater services and support. To better understand their need gaps, we assessed function, mobility, mood, and caregiver status using a mailed needs assessment questionnaire to 1112 HNHR Veterans. Among the 341(30.7%) respondents, they were primarily 274(80.4%) Non-Hispanics; 210(61.6%) Whites, and 119(34.9%) Black or African Americans; average age was 69.5±9.6 years old; 310(90.4%) had ≥high school education. The average Barthel ADL score was 81.5±22.8 and average Lawton IADL score was 5.8±2.2. Walking or balance issues were present among 260(75.8%), 227(66.2%) said they use an assistive device, and 167(48.7%) had suffered ≥1 fall, 43(12.5%). Regarding depression, 117(34.3%) screened positive (PHQ2 score≥3). These were significantly younger (66.7±9.1) than those who did not (70.8±9.3, p≤0.01). They were also significantly lower functioning (5.37±2.1 vs.6.38±2 Lawton IADL score, p≤0.01), more dependent (77.8±23.1 vs 86±19.2 Barthel ADL score, p≤0.01). We also observed significant differences in their telephone contact with family (never to once/week) [35(29.9%) vs. 27(13.4%), (p≤0.01)]; in meeting with friends or relatives ≥3times a week [12(10.3%) vs. 69(34.3%), (p≤0.01)]; and in likelihood of attending meetings with clubs or other organizations [94(80.3%) vs. 138(68.7%), p=0.040]. Detecting depression is a priority among HNHR Veterans. There is an urgent need to devise viable strategies to offer interventions that incorporate mental health needs and reduce social isolation, potentially addressing mobility, function, and transportation.

